# Integrative Analysis of Omics Data Reveals Regulatory Network of *CDK10* in Vitiligo Risk

**DOI:** 10.3389/fgene.2021.634553

**Published:** 2021-02-17

**Authors:** Minglong Cai, Tao Yuan, He Huang, Lan Gui, Li Zhang, Ziyuan Meng, Wenjuan Wu, Yujun Sheng, Xuejun Zhang

**Affiliations:** ^1^Department of Dermatology, The First Affiliated Hospital of Anhui Medical University, Hefei, China; ^2^Institute of Dermatology, Anhui Medical University, Hefei, China

**Keywords:** vitiligo, mendelian randomization, genome wide association study, expression quantitative trait locus, methylation quantitative trait loci

## Abstract

Vitiligo is a multifactorial polygenic disorder, characterized by acquired depigmented skin and overlying hair resulting from the destruction of melanocytes. Genome-wide association studies (GWASs) of vitiligo have identified approximately 100 genetic variants. However, the identification of functional genes and their regulatory elements remains a challenge. To prioritize putative functional genes and DNAm sites, we performed a Summary data-based Mendelian Randomization (SMR) and heterogeneity in dependent instruments (HEIDI) test to integrate omics summary statistics from GWAS, expression quantitative trait locus (eQTL), and methylation quantitative trait loci (meQTL) analysis of large sample size. By integrating omics data, we identified two newly putative functional genes (*SPATA2L* and *CDK10*) associated with vitiligo and further validated *CDK10* by qRT-PCR in independent samples. We also identified 17 vitiligo-associated DNA methylation (DNAm) sites in Chr16, of which cg05175606 was significantly associated with the expression of *CDK10* and vitiligo. Colocalization analyses detected transcript of *CDK10* in the blood and skin colocalizing with cg05175606 at single nucleotide polymorphism (SNP) rs77651727. Our findings revealed that a shared genetic variant rs77651727 alters the cg05175606 as well as up-regulates gene expression of *CDK10* and further decreases the risk of vitiligo.

## Introduction

Vitiligo is an autoimmune disease characterized by acquired depigmented skin and overlying hair, with a prevalence of approximately 0.4% in the European population (Zhang et al., 2016). It is reported that patients with vitiligo are associated with multiple comorbid autoimmune diseases and have a reduced risk of several tumors including melanoma, non-melanoma skin cancer, and internal malignancies (Paradisi et al., 2014; Dahir and Thomsen, 2018; Bae et al., 2019). The pathogenesis of vitiligo remains elusive. Important theories including the autoimmune hypothesis, reactive oxygen species model, and cellular alterations accounting for the destruction of melanocytes (Mohammed et al., 2015). Vitiligo is a multifactorial disease involved in both genetic and environmental factors. Previous studies in families or twins have reported that vitiligo has a strong genetic predisposition, with estimated heritability ranged from 50 to 80%(Hafez et al., 1983; Das et al., 1985; Arcos-Burgos et al., 2002; Zhang et al., 2004). Over decades, many genetic studies (such as candidate gene analyses, linkage studies, etc.) have been performed to identify genetic factors for vitiligo. Since 2008, genome-wide association studies (GWASs) have been applied in vitiligo and discovered approximately 100 genetic variants according to the GWAS catalog^1^.

Genome-wide association studies are the current gold standard for detecting genetic associations, it usually defines a gene that nearest to the top GWAS signals as the causal gene. However, it remains unclear whether these genes are functionally relevant to vitiligo. Indeed, most of the vitiligo-associated variants were reside in the non-coding regions (Spritz and Andersen, 2017), implying these variants contribute risk to vitiligo through modulating the expression of nearest or distal genes rather than disturb the structure of proteins. Moreover, DNA methylation (DNAm) is a major epigenetic modification that is mostly associated with transcriptional expression (Bogdanović and Lister, 2017). It can be served as a mediator of variant-gene expression. One of the basic hypotheses for the mechanism of complex disease (i.e., vitiligo) is variants regulate gene expression through DNAm, and further contribute to disease.

With the availability of GWAS, expression quantitative trait locus (eQTL), and methylation quantitative trait loci (meQTL) data, several approaches for integrating omics (such as genetic, transcriptomic, and epigenetic, etc.) data have been applied to prioritize functional genes and their regulatory network (He et al., 2013; Giambartolomei et al., 2014; Gamazon et al., 2015; Gusev et al., 2016; Wu et al., 2018). It is important because key factors in the regulatory network of diseases may be served as biomarkers for molecular typing of disease and designing new drugs. Summary data-based Mendelian Randomization (SMR) and heterogeneity in dependent instruments (HEIDI) is a mendelian randomization (MR) method that uses summary-level data to test if an exposure (i.e., gene expression) and outcome (i.e., trait) are associated because of a shared causal variant. Evidence show SMR and HEIDI is an unbiased method with the property that exposure-outcome associations identified in the analysis are free of non-genetic confounders. Moreover, SMR and HEIDI have the ability to distinguish a pleiotropic model from a linkage model comparing to most of the other methods (Zhu et al., 2016). In this study, we applied the SMR and HEIDI method to prioritize the functionally relevant genes and their regulatory elements (DNAm sites) by integrating summary statistics from vitiligo GWAS, eQTL, and meQTL data, and further validated our results by qRT-PCR and colocalization analyses.

## Materials and Methods

### Overview of Methodology

**FIGURE 1:**
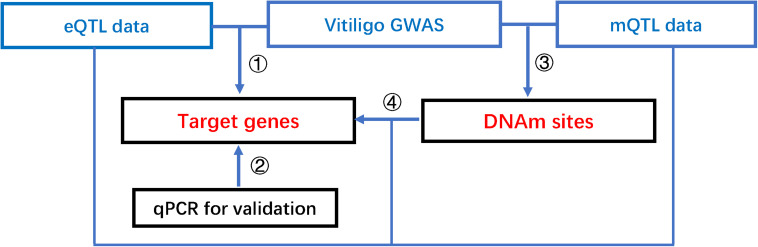
The pipeline of our integrative analysis of vitiligo. Integrative analysis of mQTL, eQTL, and vitiligo GWAS data to prioritize DNAm sites, putative functional genes, shared causal variants, and reveal a possible regulatory mechanism that the effect of genetic variants on vitiligo susceptibility is mediated by altering gene expression through DNAm.

This study integrated summary statistics from GWAS, eQTL, and meQTL data to identify vitiligo-associated genes and DNAm sites, and further revealed regulatory networks between functional elements. Our pipeline consists of four steps as follows (Figure 1): First, we integrate vitiligo GWAS and eQTL data to test the associations of gene expression with vitiligo. Next, for putative functional genes identified above, we perform qRT-PCR to confirm our results. Third, we integrate vitiligo GWAS and meQTL data to test the association of DNAm sites with vitiligo. Last, we integrate eQTL data and meQTL data to test the associations of target genes with DNAm sites of vitiligo susceptibility, and further conduct a colocalization analysis to identify shared causal variants across vitiligo, gene expression, and methylation.

### Summary Level Data

All summary statistics are listed in Table 1.

**TABLE 1:** Description of summary-level statistics.

Data source	Sample	Number of probes/variants	Vitiligo patients	Healthy controls	Population
GWAS	Blood	9,068,712 variants	2,853	37,405	European
Westra eQTL	Blood	5,016 probes	/	5,311	European
CAGE eQTL	Blood	7,136 probes	/	2,765	European
GTEx eQTL	Blood	3,725 probes	/	338	European
GTEx eQTL	Sun-exposed skin	5,526 probes	/	302	European
GTEx eQTL	Not sun-exposed skin	4,417 probes	/	196	European
eQTLGen	Blood	13,164 probes	/	31,684	European
mQTL	Blood	73,448 probes	/	1,980	European

The GWAS dataset was from the largest GWAS meta-analysis for vitiligo to date consisted of 2,853 vitiligo cases and 37,405 healthy control of European ancestry. After quality control (described elsewhere) (Jin et al., 2016), 9068712 variants remained for the following analysis. Six eQLT data used in our study were from four studies of European ancestry. The first one (Westra eQTL) was composed of blood samples from 5,311 healthy individuals (Westra et al., 2013). The second one (CAGE eQTL) has a finer single nucleotide polymorphism (SNP) coverage than the Westra eQTL with blood samples from 2,765 healthy individuals (Lloyd-Jones et al., 2017). The third one was from the GTEx project (used data from whole blood, sun-exposed skin, and not sun-exposed skin samples) composed of 80–491 individuals (Aguet et al., 2017). The last one (eQTLGen) was from the eQTLGen Consortium composed of 31,684 individuals (Võsa et al., 2018). The meQTL summary data was from a methylation wide meta-analysis conducted by McRae et al. (2018) which enrolled blood samples from a total of 1,980 healthy individuals of European ancestry. All eQTL and meQTL summary data include only non-MHC (major histocompatibility complex) gene probes.

### SMR and HEIDI Analysis

The method includes SMR and HEIDI test. SMR applies the principles of MR to integrate summary-level data (such as genetic, transcriptomic, and epigenetic, etc.) to test the associations between an exposure (i.e., transcript and DNAm) and an outcome (i.e., vitiligo) due to a shared variant at a locus. It integrates omics data of independent samples so that the statistical power can be boosted by orders of magnitude. The HEIDI test was conducted to distinguish pleiotropy (i.e., exposure and outcome are associated owing to a single shared genetic variant) from linkage (i.e., two variants in linkage disequilibrium affecting exposure and outcome independently). We performed SMR and HEIDI analyses considering gene expression as the exposure and vitiligo as the outcome to prioritize target genes in two steps. In step one, we ran SMR and HEIDI analysis to test the associations of gene expression with vitiligo by integrating vitiligo GWAS and each of five eQTL data with a relatively small sample size (Westra eQTL, CAGE eQTL, and three GTEx eQTL). For novel vitiligo-associated genes identified above, we re-ran the SMR and HEIDI analysis and integrated the GWAS with the largest eQTL data from the eQTLGen dataset (Võsa et al., 2018) to verify our results, in a second step. Threshold levels of significance for SMR tests were adjusted for multiple comparisons by Bonferroni correction (*P*_SMR_ < 0.05/number of gene probes in each eQTL analysis). Genes with *P*_HEIDI_ < 0.01 were considered as linkage and removed.

To identify DNAm sites mapping to both target gene and vitiligo, we also performed SMR and HEIDI analyses considering DNAm as the exposure and gene expression/vitiligo as the outcome in two steps. In step one, we ran SMR and HEIDI analysis to test the associations of DNAm sites with vitiligo by integrating GWAS and the *cis-*meQTL data. In step two, we re-ran SMR and HEIDI analysis to test the associations of DNAm sites with transcript by integrating meQTL and eQTLGen dataset. Threshold levels of significance for SMR tests were adjusted for multiple comparisons by Bonferroni correction (0.05/number of tests). DNAm probes with *P*_HEIDI_ < 0.01 were considered as linkage and removed.

### qRT-PCR for Validation

A total of 10 vitiligo patients and 10 age-matched healthy controls were included for independent validation. All participating individuals signed the informed consent. The study was approved by the ethic committee of the First Affiliated Hospital of Anhui Medical University. 4 ml peripheral blood were collected to extract RNA for qRT-PCR. Gene expression levels were calculated using theΔΔC_T_ method and were normalized to the expression levels of housekeeping gene-*GAPDH*. Differential gene expression analysis was performed by using *t*-test, using a *P*-value of 0.05 as the cutoff for statistical significance.

The primer of *CDK10* was below:

Forward 5′-GACCTGAAGGTTTCCAAC-3′,
Reverse 5′- ACATGTCGATGCTGGTGG-3′.
The primer of *SPATA2L* was below:
Forward 5′-TTCTCCTTCCTCTCTCTG-3′,
Reverse 5′-ACAGACCTATAGGCTGAG-3′.
The primer of reference gene (GAPDH) was below:
Forward 5′-CTTCATTGACCTCAACTACATG-3′,
Reverse 5′- CTCGCTCCTGGAAGATGGTGAT-3′.

### Colocalization Analysis

Colocalization analysis was conducted by HyPrColoc (Foley et al., 2019), which implements an efficient deterministic Bayesian algorithm to detect colocalization across multiple diseases/traits. We performed the colocalization analysis and included vitiligo GWAS, transcript of the target gene in eQTLGen and GTEx sun-exposed skin eQTL data, and DNAm sites in meQTL data. We refer to *P*_R_ as the regional association probability that all the traits share an association with one or more variants within the region and *P*_A_ as the alignment probability that the shared associations between all traits are owing to a single shared putative causal variant. The posterior probability of full colocalization (PPFC, PPFC = *P*_R_*P*_A_) represents the posterior probability that all traits share a causal variant within that region. Clusters are identified when both *P*_R_ > 0.9 and *P*_A_ > 0.9 (PPFC > 0.81) are satisfied. We refer to prior.1 as the probability that a variant is associated with a single trait and (1- prior.2) is the prior probability that a variant is associated with an additional trait given that it is associated with one trait. In the colocalization analysis, we set prior.1 = 1 × 10^–4^, and prior.2 = 0.98. Finally, we plotted a heatmap to assess the stability of clusters by specifying the parameters as follows: *P*_A_ = *P*_B_ = 0.6, 0.7, 0.8, and 0.9, piror.1 = 1 × 10^–4^, and prior.2 = 0.98, 0.99, and 0.995.

## Results

### Prioritize Vitiligo-Associated Genes

Using summary-level GWAS data from the large-scale meta-analysis of vitiligo described above, we firstly conducted the SMR analysis to test for associations of vitiligo with gene expression probes by integrating vitiligo GWAS and each of the five eQTL data (Westra eQTL, CAGE eQTL, and three GTEx eQTL). By using the threshold of corrected *P*-value (*P*_SMR_ = 0.05/number of gene probes in each eQTL analysis), five integrative analyses of SMR have identified 22, 16, 11, 6, and 4 genes that were significantly associated with vitiligo, respectively. We further applied the HEIDI method to distinguish pleiotropy from linkage, remaining 15, 11, 9, 5, and 4 genes (27 genes are unique) with a *P*_HEIDI_ > 0.01 (Supplementary Table 1). Among these genes, 13 genes were reported by previous vitiligo GWAS studies and the other 14 genes were distant from the top GWAS associated variants. To obtain more conservative results, we supposed that the integrative analysis of GWAS with five eQTL are five independent SMR and HEIDI test, which could be used for mutual validation. Only genes that passed more than two SMR and HEIDI tests were defined as newly identified genes. With this method, we determined two newly vitiligo-associated genes, *CDK10* and *SPATA2L. CDK10* was identified by SMR and HEIDI test for CAGE eQTL, GTEx sun exposure skin eQTL, and GTEx blood eQTL data. *SPATA2L* was identified by SMR and HEIDI test for Westra eQTL and GTEx blood eQTL data (Table 2). We further re-ran the SMR and HEIDI analysis and included the largest eQTL data from the eQTLGen dataset (Võsa et al., 2018) in addition to vitiligo GWAS, which confirmed that *CDK10* was associated with vitiligo (*b*_SMR_ = 0.424, *P*_SMR_ = 1.26 × 10^–8^, *P*_HEIDI_ = 0.15). However, *SPATA2L* failed to pass HEIDI test (*b*_SMR_ = −0.483, *P*_SMR_ = 2.09 × 10^–7^, *P*_HEIDI_ = 0.004, Supplementary Table 1). Given the direction of transcripts effect on vitiligo, *CDK10* was up-regulated in both the blood and skin lesion of vitiligo (*b*_SMR_ > 0). Therefore, SMR and HEIDI analyses have identified two novel genes and 13 known genes, of which *CDK10* was confirmed by integrating GWAS and eQTLGen dataset.

**TABLE 2:** Identification of 15 genes by the SMR and HEIDI analysis for vitiligo susceptibility.

Chr	Gene	Top *cis-*eQTL	*b*_SMR_^2^	Standard Error	*P*_SMR_^3^	*P*_HEIDI_^4^
4	PPP3CA	rs2583389	0.197	0.043	4.12E-06	7.64E-01
10	CASP7	rs3814231	0.585	0.102	1.03E-08	6.24E-01
11	FADS1	rs968567	−0.470	0.099	2.00E-06	3.09E-01
11	FADS2	rs968567	−0.63	0.138	4.35E-06	5.87E-01
15	HERC2	rs2016277	0.511	0.078	6.12E-11	2.50E-01
16	**SPATA2L^1^**	**rs648548**	**−0.824**	**0.169**	**1.07E-06**	**3.95E-02**
16	DEF8	rs8063761	−1.790	0.326	3.90E-08	1.27E-01
19	IRF3	rs7251	−0.366	0.070	1.42E-07	2.56E-02
22	TEF	rs9611565	−1.099	0.208	1.30E-07	1.23E-01
12	SUOX	rs7302200	−0.866	0.128	1.30E-11	2.10E-01
14	GZMB	rs2236337	−0.269	0.044	1.22E-09	1.38E-01
16	**CDK10^1^**	**rs77651727**	**0.566**	**0.108**	**1.73E-07**	**4.29E-01**
20	ASIP	rs62209647	−0.750	0.134	2.30E-08	4.09E-01
12	RPS26	rs1131017	0.192	0.025	2.54E-14	2.77E-02
21	UBASH3A	rs1893592	−1.14641	0.203846	1.87E-08	2.42E-01

*^1^Two genes were newly identified and marked in bold. ^2^The estimated effect size of gene expression on vitiligo from the SMR method. ^3^P-value from the SMR test. ^4^P-value from the HEIDI test.*

### CDK10 Was Validated by qPCR

**FIGURE 2:**
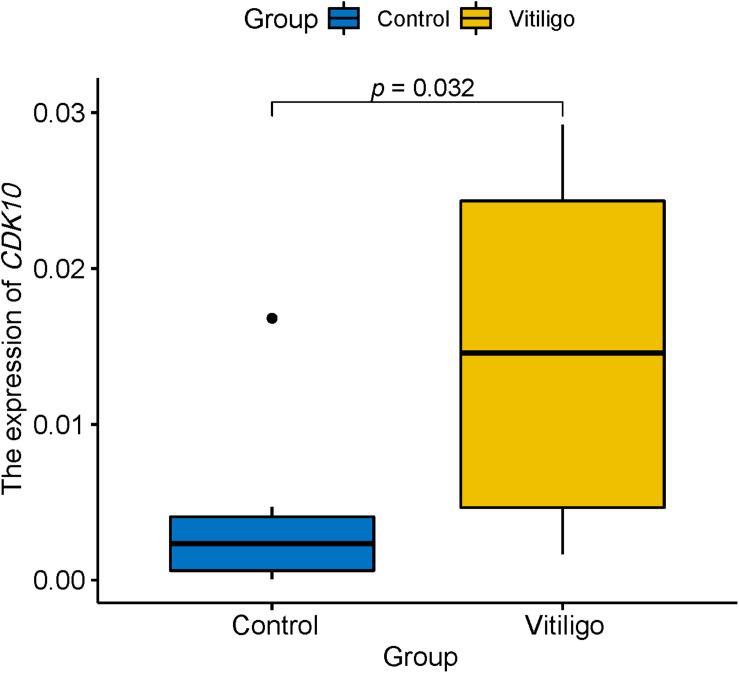
qRT-PCR showed the expression of *CDK10* is significantly increased in the blood of vitiligo relative to control.

We further included the blood samples of 10 vitiligo patients and 10 healthy control and performed qRT-PCR to verify two newly identified genes. There is no significant difference (*p* = 0.25) regarding age between patients with vitiligo (29.9 ± 4.65) and healthy control (32.5 ± 5.06). Differential gene expression analysis confirmed that mRNA level of *CDK10* was significantly increased in the blood of vitiligo (*p* = 0.032, Figure 2). However, we were unable to quantify *SPATA2L* because of its low expression level in the blood. These findings suggested that *CDK10* may functionally relevant to vitiligo.

### Plausible Mediation Mechanism for CDK10

**FIGURE 3:**
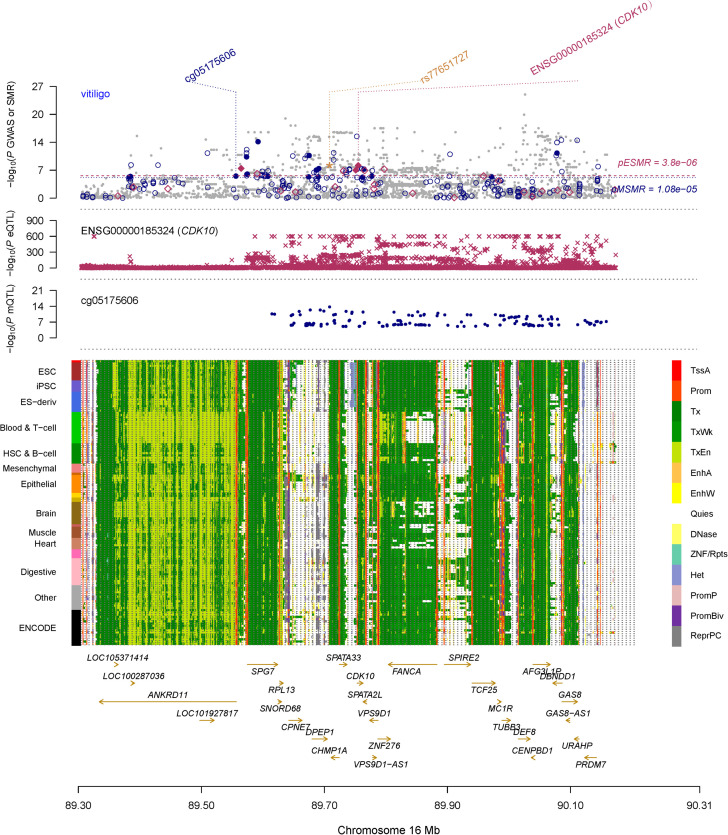
Prioritizing *CDK10* and cg05175606 for vitiligo. The top plot shows –log10(*P*-value) of SNPs from GWAS for vitiligo. The red diamonds and blue circles represent –log10(*P*-values) from SMR tests for associations of vitiligo with gene expression and DNAm probes, respectively. The solid circles and diamonds are the probes not rejected by the HEIDI test, the yellow star indicates the top-associated variant rs77651727 in the eQTL analysis of the *CDK10* and the meQTL analysis of the cg05175606. The second plot shows –log10(*P*-values) from the eQTLGen dataset for associations of SNPs with gene expression probe ENSG00000185324 (tagging CDK10). The third plot shows –log10(*P*-values) from meQTL study for associations of the SNPs with DNAm probes (cg05175606). The bottom plot shows 14 chromatin state annotations (indicated by colors) of 127 samples from REMC for different primary cells and tissue types (rows). TSSA, active TSS; Prom, promoter, Tx, active transcription; TxWk, weak transcription; TxEn, transcribed and regulatory promoter/enhancer; EnhA, active enhancer; EnhW, weak enhancer; DNase, primary DNase; ZNF/Rpts, ZNF genes and repeats; Het, heterochromatin; PromP, poised promoter; PromBiv, bivalent promoter; ReprPC, repressed PolyComb; Quies, quiescent/low.

To identify DNAm sites mapping to both vitiligo and transcript of *CDK10*, we firstly performed SMR and HEIDI analysis to test the associations of DNAm sites with vitiligo by integrating the GWAS and meQTL data. We only included DNAm probes in Chr16 where *CDK10* is located. Threshold levels of significance for SMR tests were adjusted for multiple comparisons by Bonferroni correction (*p* < 1.08 × 10^–5^ after correcting for 4,626 DNAm probes in chr16). We found 17 DNAm sites associated with vitiligo (*P*_SMR_ < 1.08 × 10^–5^ and *P*_HEIDI_ > 0.01, Supplementary Table 2A). Then, we re-ran SMR and HEIDI analysis to test the associations of all DNAm sites with all transcript of genes in chr16 by integrating meQTL and eQTL data. The gene expression probes used in this analysis were retained from eQTLGen data because it has the best power. Threshold levels of significance for SMR tests were adjusted for multiple comparisons by Bonferroni correction (*P*_SMR_ < 2.09 × 10^–7^ after correcting for 238,766 tests). We finally identified cg05175606 was involved in relatively distal regulation of gene expression of *CDK10* (*P*_SMR_ = 3.8 × 10^–14^, *P*_HEIDI_ > 0.01, Supplementary Table 2B) and associated with vitiligo (*P*_SMR_ = 4.67 × 10^–7^, *P*_HEIDI_ > 0.01, Supplementary Table 2A). Therefore, cg05175606 was mapped both *CDK10* and vitiligo, and located at the promoter region according to the chromatin state annotations from the Roadmap Epigenomics Mapping Consortium (REMC) (Lloyd-Jones et al., 2017) reference samples (Figure 3).

### Transcript of CDK10 Colocalizing with cg05175606 at rs77651727

**FIGURE 4:**
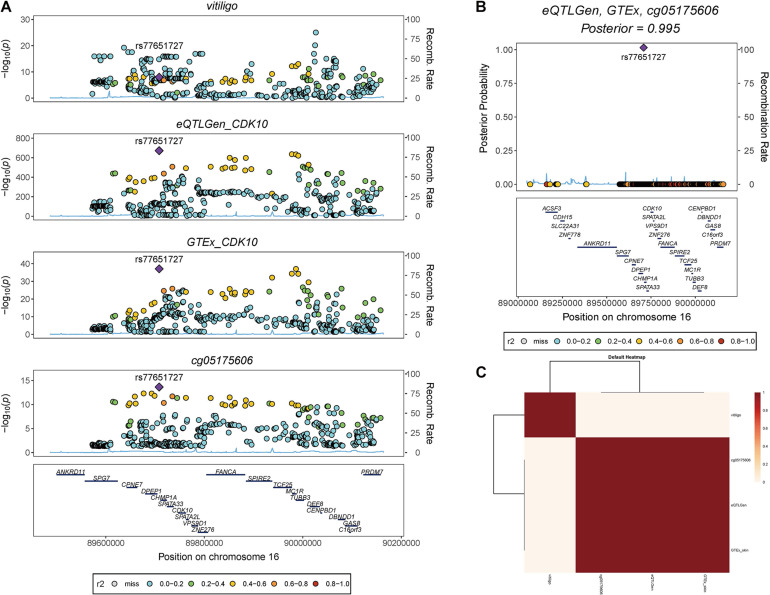
Colocalization analysis of vitiligo, transcript of *CDk10*, and cg05175606. **(A)** Stacked association plots of vitiligo with the transcript of *CDK10* in eQTLGen and GTEx sun-exposed skin eQTL data, and cg05175606 in meQTL data. HyPrColoc detected rs77651727 is the top-associated variant in eQTL and meQTL data. The variant is also associated with vitiligo at a genome-wide significant level. **(B)** HyPrColoc identified rs77651727 as a candidate causal variant explaining the shared association signal between the transcript of *CDK10* and cg05175606, i.e., rs77651727 explained 100% of the posterior probability of colocalization. **(C)** HyPrColoc sensitivity analysis shows the transcript of *CDK10* in two eQTL analysis and cg05175606 form a very strong cluster of colocalized traits.

We also found rs77651727 is the top associated SNP in both the eQTL analysis of the *CDK10* (*b*_eQTL_ = −0.945, *P*_eQTL_ < 1 × 10^–100^) and the meQTL analysis of the cg05175606 (*b*_meQTL_ = 0.427, *P*_meQTL_ = 1 × 10^–14^). To investigate if this variant is the shared genetic association across vitiligo, transcript of *CDK10*, and cg05175606. We used HyPrColoc to perform a colocalization analysis in pre-defined independent linkage disequilibrium blocks spanning the Chr16 (Berisa and Pickrell, 2016). HyPrColoc identified transcript of *CDK10* in eQTLGen and GTEx skin eQTL dataset colocalizing with cg05175606 at rs77651727 (PPFC = 0.995 of which 100% is explained by the variant rs77651727; Figure 4). Although vitiligo was not colocalizing with QTL at this SNP, the SNP was associated with vitiligo at a genome-wide significant level (*P*_GWAS_ = 9.51 × 10^–10^) and the minor T allele of rs77651727 was found to has a protective effect on vitiligo (*b*_GWAS_ < 0). Moreover, rs77651727 is located at the enhancer region across multiple tissues (Supplementary Figure 1A; Kundaje et al., 2015). The SNP-association signal is significant and consistent across GWAS, eQTLGen, GTEx skin eQTL, and mQTL implying a plausible mechanism in which rs77651727 at the enhancer region alters the cg05175606 and up-regulates the expression of the *CDK10* and therefore decreases the risk of vitiligo (Supplementary Figure 1B).

## Discussion

To our knowledge, ours is the first omics study of vitiligo. By integrative analysis of summary-level data from vitiligo GWAS, six eQTL, and one meQTL studies, we prioritize a novel target gene and its regulatory elements. We also found a candidate shared causal variant (rs77651727) across vitiligo, transcript of *CDK10*, and cg05175606. Our findings indicate a plausible mechanism whereby the effect of genetic variants on vitiligo is mediated by genetic regulation of target gene as well as DNA methylation.

In this study, the SMR and HEIDI method was first applied to prioritize functional relevant genes of vitiligo susceptibility by integrating GWAS and each of five eQTL data. We found a total of 15 vitiligo-associated genes, which is of more biological interest than the GWAS results because genes identified by SMR and HEIDI were differentially expressed between vitiligo and control. Among 15 genes, 13 of them were reported by previous GWAS (Jin et al., 2010, 2012, 2016; Tang et al., 2013). The other two newly identified genes, *CDK10* and *SPATA2L*, could be potential functional relevant genes. We further performed SMR and HEIDI by combing GWAS with the largest eQTL data from eQTLGen Consortium, which verified *CDK10* as a vitiligo-associated gene. qPCR also confirmed that *CDK10* is up-regulated in the blood of vitiligo patients relative to healthy controls. Of note, previous GWASs of vitiligo have identified rs9926296 and rs4268748 as susceptibility variants, and both SNPs were mapped to *MC1R*, which was finally reported as a vitiligo-associated gene (Jin et al., 2012, 2016). However, both variants were not associated with the expression of *MC1R* but significantly associated with the expression of *CDK10* across multiple tissues in the GTEx dataset (Supplementary Table 3). Hence, we have good reason to believe that *CDK10* is a potential causal gene. *CDK10* is a CDK1-associated kinase that regulates cell cycle progression from the G2 to the M phase (Li et al., 1995), which is known to be essential for cell cycle progression. It is regarded as a key candidate tumor suppressor in multiple cancer types, including melanoma, hepatocellular carcinoma, and gastric cancer, etc., (Zhong et al., 2012; Ransohoff et al., 2017; You et al., 2018). In this study, the finding of up-regulation of *CDK10* expression in vitiligo support the evidence that patients with vitiligo have a reduced risk of overall internal malignancies (Bae et al., 2019), melanoma, and non-melanoma skin cancer (Paradisi et al., 2014).

By integrating vitiligo GWAS and meQTL data, we prioritize 17 vitiligo-associated DNAm in Chr16, with 12 hypomethylated (*b*_SMR_ < 0) and 5 hypermethylated (*b*_SMR_ > 0) in vitiligo relative to control. Then we discovered that cg05175606 was mapped to both *CDK10* and vitiligo. The negative effect of the DNAm site on gene expression indicates a repressing role of cg05175606 on the expression of *CDK10*. Of note, *CDK10* is a distal gene beyond 200 kb distance from cg05175606, providing a caveat that DNAm do not always map to the nearest target genes (Wu et al., 2018). For all genes in chr16, we found *ANKRD11* is the nearest gene to cg05175606 (only 880 bp away from the gene). Although cg05175606 is in the promoter region of *ANKRD11*, it is not mapped to the nearest gene (*ANKRD11, P*_SMR_ = 1.53 × 10^–2^) but to the relatively distal *CDK10* (*P*_SMR_ = 3.8 × 10^–14^), *SPG7* (*P*_SMR_ = 1.59 × 10^–9^), and *SPATA2L* (*P*_SMR_ = 3.32 × 10^–8^, Supplementary Table 2B), not supporting the hypothesis that the distal associations are mediated through the nearest genes.

To further reveal the causal relationship between cg05175606 and *CDK10*, we re-ran the SMR and HEDI analysis considering gene expression as the exposure and DNAm as the outcome, which identified the transcript of *CDK10* also had a repressing effect on cg05175606 (*b*_SMR_ = −0.45, *P*_SMR_ = 3.8 × 10^–14^). This makes sense because rs77651727 is the top associated variant in both the eQTL analysis of *CDK10* and meQTL analysis of cg05175606, and therefore both SMR and HEIDI analyses use this variant as the instrument. Hence, the association between the transcript of *CDK10* and cg05175606 is driven by the shared variant rs77651727, and causal relationship between *CDK10* expression and cg05175606 is not clear because they both affect each other.

To determine if genetic variant rs77651727 was shared across vitiligo, transcript of *CDK10* in the blood and skin tissue, and cg05175606, we performed a colocalization analysis and detected that transcript of *CDK10* in the blood and skin tissue were colocalized with cg05175606 at rs77651727. We noted that vitiligo was not colocalized with transcript of *CDK10* and cg05175606. But SMR and HEIDI analysis has identified the transcript of *CDK10* and cg05175606 were associated with vitiligo due to the shared variant rs77651727. This variant is the top marker in the eQTL analysis of *CDK10* and meQTL analysis of cg05175606, implicating a more important biological meaning of rs77651727 than others. Moreover, colocalization analysis by using HyPrColoc is under the assumption of at most one causal variant per trait, which means a secondary causal variant (be in weak LD with the top marker) is undetectable. rs77651727 is in weak LD with the reported top variant rs4268748 (*r*^2^ = 0.167) and reached the genome-wide significant level in vitiligo GWAS (*p* = 9.51 × 10^–10^). It is reasonable that HyPrColoc regarded rs4268748, but not rs77651727, as the causal variant, and therefore vitiligo was not colocalized with other QTLs. We also re-ran HyPrColoc to access the colocalization across all traits (specify the parameter: bb.alg = FALSE) and found the PR equal to 1, indicating all traits share an association with one or more variants within the region despite no causal variant was identified. Based on all these evidences, we speculate that rs77651727 is a candidate causal variant of vitiligo susceptibility, and the protective role of rs77651727 on vitiligo is mediated by altering the cg05175606 as well as up-regulating distal gene expression of *CDK10.*

There are limitations to our study. First, the samples size for qRT-PCR validation are small; Second, we have not performed qRT-PCR in skin samples despite a higher mRNA level of *CDK10* in the skin than that in the blood, which needs further validation. Moreover, we identified cg05175606 was not mapped to the nearest gene but to the relatively distal *CDK10, SPG7*, and *SPATA2L.* However, no evidence shows an interactive effect among these distal genes, there is a possibility that *CDK10* was affected by other distal genes (i.e., *SPG7* and *SPATA2L*) through cg05175606. Further studies will be required for elucidating the regulatory network among these elements and the functional mechanisms of *CDK10* in the pathogenesis of vitiligo.

In conclusion, by integrating genomic, transcriptomic, and epigenomic data, we prioritized one newly functional gene and its regulatory elements. rs77651727, cg05175606, and *CDK10* constructed a regulatory network involving cell proliferation, differentiation, and death, which might alter the risk of vitiligo.

## Data Availability Statement

The LD information was computed using the phased haplotypes from the 1000 Genomes study (http://www.internationalgenome. org/). The summary-level data of vitiligo GWAS is available in https://www.ebi.ac.uk/gwas/. Westra eQTL, CAGE eQTL, and GTEx eQTL summary data were downloaded from https://cnsgenomics.com/software/smr/#DataResource. SMR-formatted cis-eQTLs summary data is contributed by eQTLGen (http://www.eqtlgen.org/cis-eqtls.html).

## Ethics Statement

The studies involving human participants were reviewed and approved by The First Affiliated Hospital of Anhui Medical University. The patients/participants provided their written informed consent to participate in this study.

## Author Contributions

MC, YS, and XZ: conceptualization. MC, TY, and HH: formal analysis and visualization. LZ, LG, ZM, and WW: data curation. MC: writing–original draft preparation. YS and TY: writing–review and editing. YS and XZ: funding acquisition. All authors have read and agreed to the published version of the manuscript.

## Conflict of Interest

The authors declare that the research was conducted in the absence of any commercial or financial relationships that could be construed as a potential conflict of interest.

## Footnotes

1 https://www.ebi.ac.uk/gwas/

## Supplementary Material

Supplementary Figure 1 Prioritizing *CDK10* and regulatory elements for vitiligo with a plausible regulation mechanism. **(A)** An enlarged plot for Figure 3 shows rs77651727 is located in the enhancer region across multiple tissues. **(B)** A hypothetical regulation mechanism that the protective role of rs77651727 on vitiligo is mediated by demethylation of cg05175606 and up-regulation of *CDK10* expression. TSSA, Active TSS; Prom, Promoter, Tx, Active transcription; TxWk, weak transcription; TxEn, Transcribed and regulatory Promoter/Enhancer; EnhA, Active enhancer; EnhW, Weak enhancer; DNase, Primary DNase; ZNF/Rpts, ZNF genes & repeats; Het, Heterochromatin; PromP, Poised Promoter; PromBiv, BivalentPromoter; ReprPC, Repressed PolyComb; Quies, Quiescent/Low.

Supplementary Table 1 Pleiotropic associations between genes and vitiligo.

Supplementary Table 2 Pleiotropic associations of DNAm with vitiligo and transcript.

Supplementary Table 3 Single-Tissue eQTLs for *CDK10* and MC1R from GTEx dataset.
