# Supplementary figures and images for: Integrative Analysis of Omics Data Reveals Regulatory Network of CDK10 in Vitiligo Risk
Source: Front Genet. 2021 Feb 17;12:634553. doi: 10.3389/fgene.2021.634553 (PMC7925885; doi:10.3389/fgene.2021.634553)

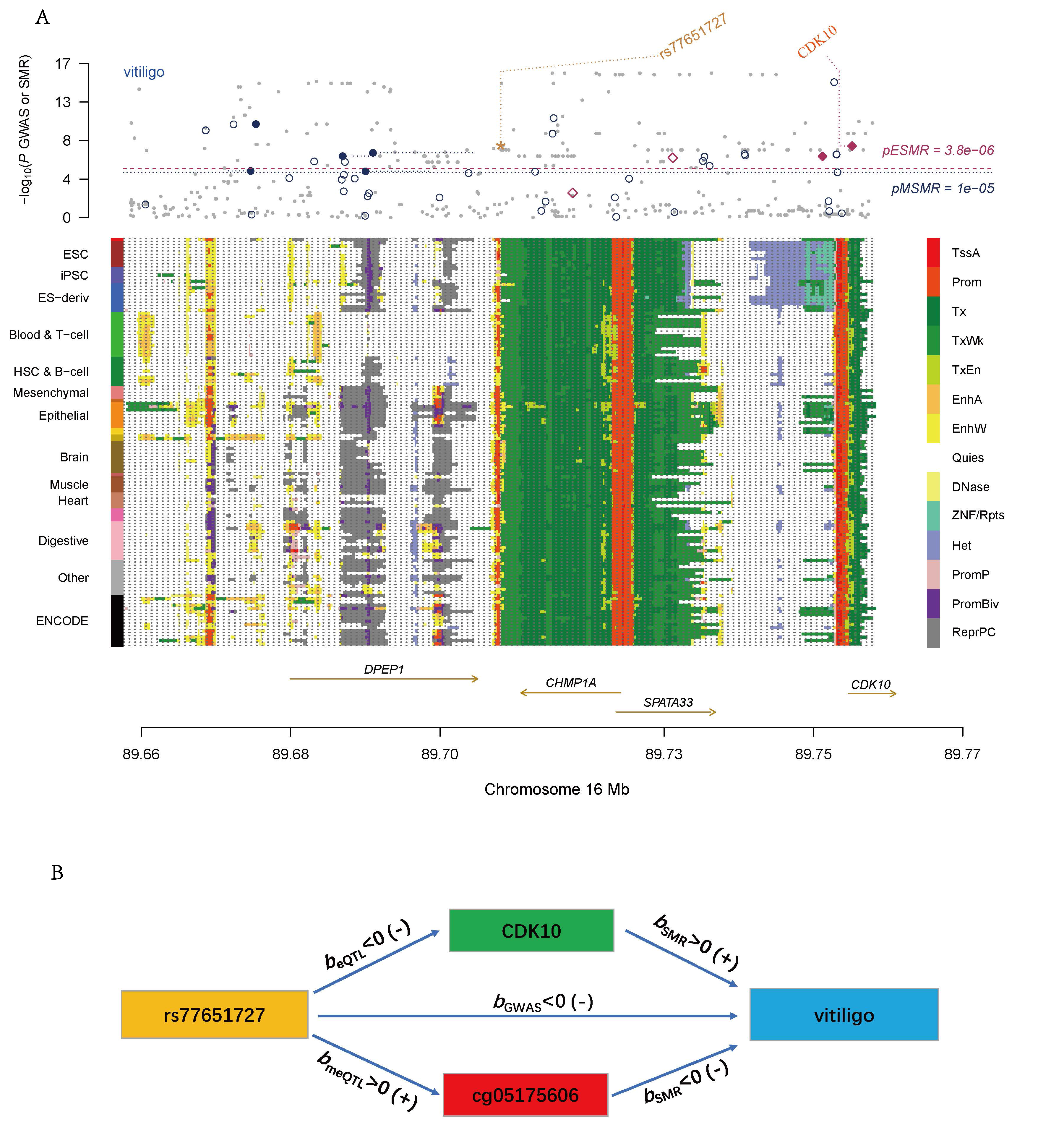

Supplement: Supplementary Figure 1 — Prioritizing CDK10 and regulatory elements for vitiligo with a plausible regulation mechanism. (A) An enlarged plot for Figure 3 shows rs77651727 is located in the enhancer region across multiple tissues. (B) A hypothetical regulation mechanism that the protective role of rs77651727 on vitiligo is mediated by demethylation of cg05175606 and up-regulation of CDK10 expression. TSSA, Active TSS; Prom, Promoter, Tx, Active transcription; TxWk, weak transcription; TxEn, Transcribed and regulatory Promoter/Enhancer; EnhA, Active enhancer; EnhW, Weak enhancer; DNase, Primary DNase; ZNF/Rpts, ZNF genes & repeats; Het, Heterochromatin; PromP, Poised Promoter; PromBiv, BivalentPromoter; ReprPC, Repressed PolyComb; Quies, Quiescent/Low. [file Image_1.TIF]
